# Proteolytic Cleavage of Apolipoprotein E in the Down Syndrome Brain

**DOI:** 10.14336/AD.2015.1020

**Published:** 2016-05-27

**Authors:** Ryan J. Day, Katie L. McCarty, Kayla E. Ockerse, Elizabeth Head, Troy T. Rohn

**Affiliations:** ^1^Department of Biological Sciences, Boise State University, Boise, Idaho, 83725, USA; ^2^Department of Pharmacology& Nutritional Sciences, Sanders-Brown Center on Aging, University of Kentucky, Lexington, KY, 40536, USA

**Keywords:** Alzheimer’s disease, beta-amyloid, paired helical filaments, proteolysis, neurofibrillary tangles, immunohistochemistry

## Abstract

Down syndrome (DS) is one of the most common genetic causes of intellectual disability and is characterized by a number of behavioral as well as cognitive symptoms. Many of the neuropathological features of early-onset Alzheimer’s disease (AD) including senile plaques and neurofibrillary tangles (NFTs) are also present in people with DS as a result of triplication of the amyloid precursor gene on chromosome 21. Evidence suggests that harboring one or both apolipoprotein E4 (*APOE4*) alleles may increase the risk for AD due to the proteolytic cleavage of apoE4 and a subsequent loss of function. To investigate a role for the apoE proteolysis *in vivo*, we compared three autopsy groups; 7 DS with AD neuropathology cases over 40 years, 5 young DS cases without AD pathology under 40 years (YDS) and 5 age-matched control cases over 40 years by immunohistochemistry utilizing an antibody that detects the amino-terminal fragment of apoE. Application of this antibody, termed the amino-terminal apoE fragment antibody (nApoECF) revealed labeling of pyramidal neurons in the frontal cortex of YDS cases, whereas in the DS-AD group, labeling with nApoECF was prominent within NFTs. NFT labeling with nApoECF was significantly greater in the hippocampus versus the frontal cortex in the same DS-AD cases, suggesting a regional distribution of truncated apoE. Colocalization immunofluorescence experiments indicated that 52.5% and 53.2% of AT8- and PHF-1-positive NFTs, respectively, also contained nApoECF. Collectively, these data support a role for the proteolytic cleavage of apoE in DS and suggest that apoE fragmentation is closely associated with NFTs.

Down syndrome (DS) is a chromosomal disorder (Trisomy 21) that is caused by nondisjunction resulting in the triplication of the chromosome 21 in the large majority of cases and is the most commonly identified genetic cause of intellectual disability in the United States [1, 2]. In addition to several phenotypic features of DS [3-7], people with DS also exhibit mild to moderate cognitive dysfunction [8] delayed verbal short-term memory and neurobehavioral problems [9]. In addition to the phenotypic and cognitive impairments associated with this disorder, an increased risk of Alzheimer’s disease (AD) in patients with DS is also well established. In DS, the postmortem findings of senile plaques and neurofibrillary plaques (NFTs) is assumed to be caused by the overexpression of the amyloid precursor protein (APP) following triplication of chromosome 21 and accumulation of beta-amyloid. In DS, nearly all adults over 35 to 40 years of age exhibit key neuropathological changes characteristic of AD including the formation of extracellular plaques of beta-amyloid and NFTs [10-12].

Previous studies have shown a substantial increase in the cumulative incidence of dementia in adults with DS between ages 50 and 72 [13]. Risk factors that affect the age of onset of dementia in DS include harboring the *APOE4* allele as well as high levels of plasma beta-amyloid 1-42 [14]. In contrast, there is a reduced risk for the onset of dementia associated with *APOE2* allele and atypical karyotypes in DS [14]. The human *APOE* gene is polymorphic resulting in three major isoforms, apoE2, apoE3, and apoE4, which differ by single amino acid substitutions involving cysteine-arginine replacements at positions 112 and 158 [15]. Inheritance of one copy of the *APOE4* allele increases the disease risk of AD four-fold, while two copies enhances disease risk approximately ten-fold [16]. Thus, harboring the *APOE4* allele represents the most significant late-onset genetic risk factor. A recent study highlighted this risk by demonstrating that the lifetime risk of AD at the age of 85 without reference to the *APOE* genotype was 11% in males and 14% in females [17]. At the same age, this risk ranged from 51% for *APOE* 4/4 male carriers to 60% for *APOE* 4/4 female carries, consistent with a semi-dominant inheritance pattern [17]. The preponderance of evidence suggests that harboring the *APOE4* allele in DS also increases AD disease risk, although to a lower extent to what has been found in AD [18]. Additionally, studies suggest harboring the *APOE4* allele leads to earlier mortality in the DS population that is independent of the risk of dementia [19, 20]. How apoE4 increases the risk for AD is unknown, however, evidence suggests that the enhanced susceptibility of apoE4 to proteolysis as compared to E2 and E3 may play a critical role leading to loss of function including impaired cholesterol transport and beta-amyloid clearance [21]. The purpose of the current study was to investigate whether apoE proteolysis is prevalent in postmortem DS human brain sections utilizing an antibody that detects the amino-terminal fragment of apoE (herein termed, nApoECF antibody) [22]. Previous studies carried out with the nApoECF antibody demonstrated that it consistently labeled NFT’s in sporadic AD, Picks disease and vascular dementia in addition to the labeling of blood vessels and reactive astrocytes [22-24]. Our findings using the nApoECF antibody in the present study support a role for the proteolytic cleave of apoE with aging and AD in DS and suggest that apoE fragmentation is closely associated with mature NFTs.

**Table 1:** Case Demographics

Case	NPD	Sex	PMI	Age	Region	Cause of Death	APOE Genotype
**1**	Normal	F	24	46	Frontal Cortex	Multiple injuries	N/A
**2**	Normal	F	21	51	Frontal Cortex	Cardiovascular disease	N/A
**3**	Normal	M	17	57	Frontal Cortex	Arteriosclerotic cardiovascular disease	N/A
**4**	Normal	M	5	65	Frontal Cortex	Cardiac arrest	N/A
**5**	Normal	M	3	67	Frontal Cortex	Cardiomyopathy	N/A
**6**	YDS	M	24	24	Frontal Cortex	Cardiac arrhythmia	N/A
**7**	YDS	M	4	31	Frontal Cortex	Pneumonia	N/A
**8**	YDS	F	36	34	Frontal Cortex and Hippocampus	Septic shock	N/A
**9**	YDS	F	12	39	Frontal Cortex and Hippocampus	Cancer	N/A
**10**	YDS	M	5	33	Frontal Cortex and Hippocampus	Acute bronchopneumonia	N/A
**11**	DS-AD	M	6	46	Frontal cortex and Hippocampus	Cardiac respiratory failure	2/3
**12**	DS-AD	M	18	56	Frontal cortex and Hippocampus	Alzheimer’s disease	3/3
**13**	DS-AD	F	5	57	Frontal cortex and Hippocampus	Other	3/3
**14**	DS-AD	M	10	66	Frontal Cortex	Congestive heart failure	N/A
**15**	DS-AD	M	2	67	Frontal Cortex	Pneumonia	N/A
**16**	DS-AD	F	3	57	Hippocampus	Alzheimer’s disease	3/3
**17**	DS-AD	M	2.2	49	Hippocampus	Pneumonia	3/3

PMI, postmortem interval in hours; NPD, neuropathological diagnosis; YDS, young Down Syndrome; DS-AD, Down syndrome with Alzheimer’s disease pathology. For all DS-AD cases, the Braak & Braak staging was VI/C.

## MATERIALS AND METHODS

### Subjects

Autopsy brain tissue was obtained from three groups - Young DS (YDS), DS with sufficient neuropathology for AD (DS-AD) and age matched controls for the DS-AD cases. Case demographics are presented in Table 1. Fixed hippocampal tissue sections used in this study were provided by either the Institute for Memory Impairments and Neurological Disorders at the University of California, Irvine or the NIH NeuroBioBank. Approval from Boise State University Institutional Review Board was not obtained due to the exemption granted that all tissue sections were fixed and received from University of California, Irvine. Brain tissue obtained from University of California, Irvine were anonymized and never identified except by case number. Tissue donors or their next of kin provided informed signed consents to the Institute for Memory Impairments and Neurological Disorders for the use of their tissues in research (IRB 2014-1526). AD was established in DS cases based upon published consensus neuropathological criteria [25].

### Immunohistochemistry

Free-floating 40 μm-thick sections were used for bright-field immunohistochemical studies as previously described [23]. The primary antibody was visualized using brown DAB substrate (Vector Laboratories).

### Immunofluorescence Microscopy

Primary antibodies utilized included PHF-1 (mouse monoclonal, 1:1,000), AT8 (mouse monoclonal, 1:250) and nApoECF (rabbit monoclonal, 1:100). PHF-1 was a generous gift from Dr. Peter Davies (Albert Einstein College of Medicine, Bronx, NY). The AT8 antibody was purchased from Pierce, ThermoFisher Scientific Inc. (Waltham, MA). The anti-apoE4 full-length C-terminal mouse antibody was purchased from Abgent (San Diego, CA). The anti-apoE4 full-length N-terminal mouse antibody was purchased from Aviva Systems Biology Corp. (San Diego, CA). No antigen retrieval methods were employed. For double-label immunofluorescence co-localization studies, experiments were conducted as previously described [23]. Briefly, an Olympus BX60 microscope with fluorescence capability equipped with a MagnaFire SP software system for photomicrography was employed for microscopic observation and photomicrography of the DAB-labeled and fluorescent sections. The fluorescent molecules were excited with a 100-W mercury lamp. Fluorescent-labeled molecules were detected using a filter set having a 460-500-nm wavelength band pass excitation filter, a 505-nm dichroic beam splitter, and a 510-560-nm band pass emission filter.

### Confocal microscopy

Confocal immunofluorescence imaging was as previously described [23], and primary antibodies were visualized with secondary antibodies tagged with either Alexa Fluor 488 or Alexa Fluor 555 (Invitrogen, Carlsbad, CA). Images were taken with a Zeiss LSM 510 Meta system combined with the Zeiss Axiovert Observer Z1 inverted microscope and ZEN 2009 imaging software (Carl Zeiss, Inc., Thornwood, NY). Confocal Z-stack and single plane images were acquired with an Argon (488 nm) and a HeNe (543 nm) laser source. Z-stacks images were acquired using a 20x Plan-Apochromat (NA 0.8) objective, emission band passes of 505-550 nm for the detection of the nApoECF (green channel, Alexa Fluor 488) and 550-600 nm for detection of PHF-1 (red channel, Alexa Fluor 555). All images displayed are 2-D, maximal intensity projections generated acquired Z-stacks. The optical depth used varied between experiments but was in the range of 10-25 µm in the Z-plane. Single plane images were acquired with a 63x Plan-Apochromat oil-immersion objective (NA 1.4) with emission long pass of 505 nm for the detection of the nApoECF antibody (green channel, Alexa Fluor 488) and 550-600 nm for the detection of PHF-1 (red channel, Alexa Fluor 555).

### Statistical analysis

To determine significant differences between the various cohorts, an ANOVA was used to test for group differences in cell counts. IBM SPSS Statistics (Ver. 22) was used for statistical analyses with an alpha level of 0.05.

To determine the percent co-localization, a quantitative analysis was performed as described previously [23] by taking 20X immunofluorescence, overlapping images from three different fields in area CA1 in three separate DS-AD cases. Capturing was by using a 2.5x photo eyepiece, and a Sony high resolution CCD video camera (XC-77). For example, to determine the percent co-localization between nApoECF and PHF-1, photographs were analyzed by counting the number of nApoECF or PHF-1-positive NFTs alone per 20X field for each case, and the number of cells labeled with both PHF-1 and nApoECF. Data are representative of the average number (±S.D.) of each antibody alone or co-localized with both antibodies in each 20X field (3 fields total for 4 different cases). Statistical differences in this study were determined using Student’s two-tailed T-test employing Microsoft Office Excel. We used the Kolmogorov-Smirnov test of normality (SPSS Statistics) and all groups were normally distributed and thus, it was appropriate to use an ANOVA for the analysis. To determine any possible correlations between the various groups, Pearson’s coefficients were determined using Microsoft Office Excel.

**Figure 1.**
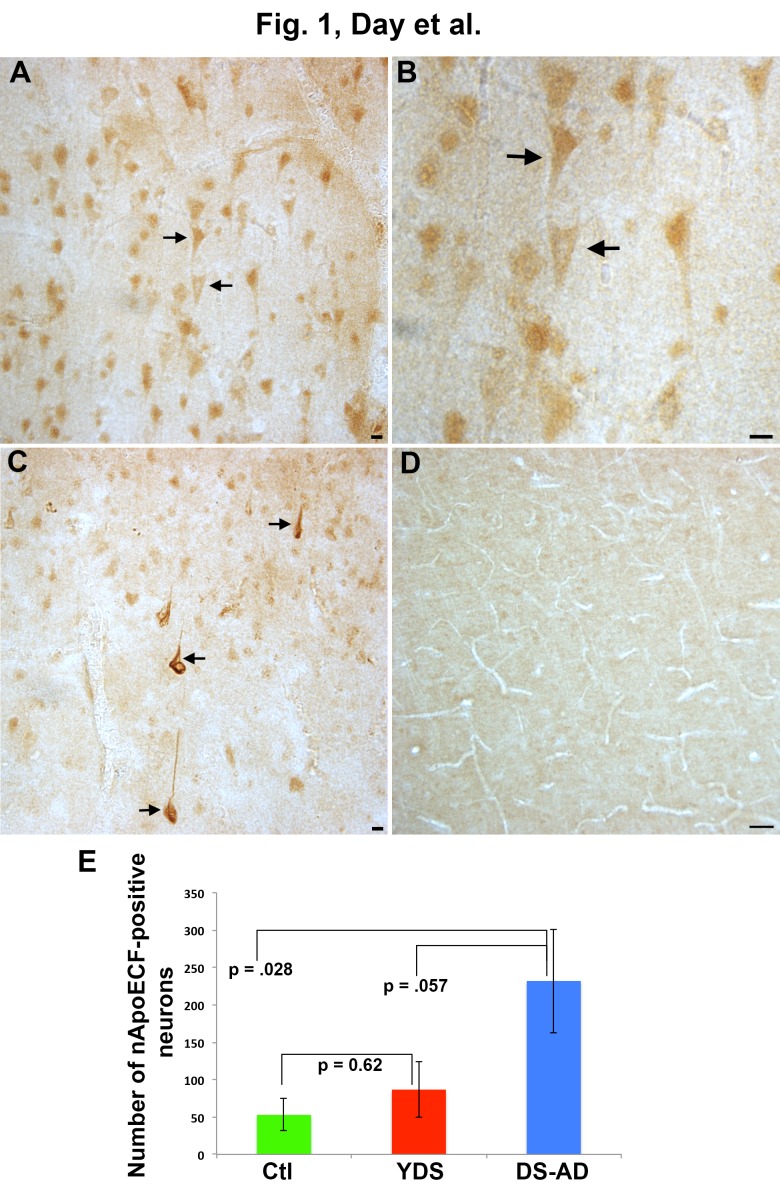
Localization of an amino-terminal fragment of apoE in the frontal cortex of Down’s syndrome (**A-B**): Application of the nApoECF antibody in frontal cortex tissue sections from YDS cases revealed labeling predominantly within pyramidal neurons (arrows, low magnification Panel A) and (arrows, high magnification Panel B). (**C**): In contrast, in DS-AD cases, in addition to neuronal staining, more mature, fibrillary NFTS were labeled with the nApoECF antibody (arrows), whereas little immunoreactivity was observed in age-matched control cases (**D**). (**E**): A least significant difference post hoc test analysis of the number of nApoECF-positive neurons and NFTs in frontal cortex indicated a significant difference between DS-AD cases and age-matched controls (p= 0.02) and YDS groups (p = 0.05). For all three groups, n = 5, ±S.E.M. All scale bars represent 10 µm except for Panel D, which represents 50 µm.

**Figure 2.**
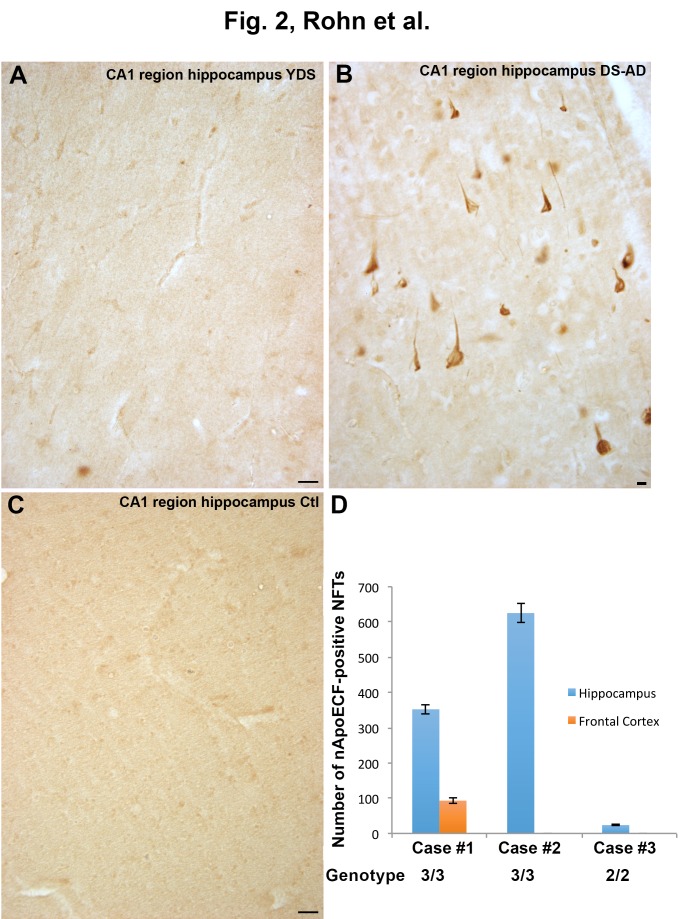
Localization of an amino-terminal fragment of apoE in the hippocampus of Down’s syndrome Application of the nApoECF antibody in hippocampal tissue sections revealed very little labeling in YDS cases (**A**) or in age-matched controls (**C**), however, strong immunolabeling of NFTs was observed in DS-AD cases (**B**). (**D**): Three DS-AD cases were quantified (±S.D.) for the number of nApoECF-positive NFTs comparing hippocampal versus frontal cortex regions. The data revealed a clear difference between the numbers of labeled NFTs between the two regions with the hippocampus giving consistently higher numbers. Case #1, #2, and #3 correspond to subjects 13, 12, and 11, respectively, as listed in Table 1. All scale bars for Panels A and C represent 50 µm and 10 µm for Panel D.

## RESULTS

### Localization of the amino terminal fragment of apoE in the frontal cortex of Down’s syndrome

To investigate a role for the apoE proteolysis in DS, an immunohistochemical study utilizing our in house nApoECF antibody was performed in fixed frontal cortex and hippocampal brain sections from 7 adult DS-AD cases, 5 YDS cases, and 5 neuropathogically normal cases. Case demographics for the DS-AD, YDS and control groups are presented in Table 1. Age at death between the control and DS-AD groups was not statistically different (57.2 ±8.96 vs. 56.9 ±7.82). As an initial step, we screened all cases for nApoECF immunoreactivity using bright field microscopy. Previous characterization of this nApoECF antibody has indicated it to be highly specific for a neoepitope ~18 kDa amino-terminal fragment of apoE [22]. This in house antibody was synthesized based upon a putative protease-cleavage site at position D172 of the full-length protein and has been extensively characterized biochemically to show that it does not react at all with the full-length form of apoE [22]. Application of this antibody to YDS frontal cortex brain sections revealed specific localization predominantly within pyramidal neurons (Figure 1A, arrows and 1B). By contrast, application of the same antibody revealed labeling of both mature fibrillary NFTs (Figure 1C arrows) as well as neuronal staining in DS-AD, while minimal immunoreactivity was observed in normal control cases (Figure 1D). Quantitative analysis of labeled neurons and NFTs in the frontal cortex indicated that there was a significant main effect of group on cell counts with controls having the fewest positive cells, the young DS having an intermediate number of cells and the DS-AD cases having the highest number of positive cells (F(2, 14)=4.08 p=0.044). A significant difference was found between the age-matched controls and DS-AD group (p= 0.02) as well as between the YDS and DS-AD group (p= 0.05) (Figure 1E).

### Localization of the amino terminal fragment of apoE in the hippocampus of Down’s syndrome

To determine if nApoECF labeling was specific to the frontal cortex similar experiments were conducted on hippocampal sections. In contrast to what was observed in frontal cortex sections, application of the nApoECF antibody to hippocampal sections revealed little to no immunoreactivity in YDS and normal control cases (Figure 2A and 2C). As observed in the frontal cortex cases, there was strong immunolabeling of NFTs as well as neuronal staining in DS-AD cases (Figure 2B). To verify the observed differences between hippocampal and frontal cortex regions three DS-AD cases were quantified for the number of nApoECF-positive NFTs (Figure 2D). The results indicated that between the two regions the hippocampus consistently had higher numbers of labeled NFTs, suggesting a regional distribution of truncated apoE. The three cases used in this experiment were subjects 11 (case #3), 12 (case #2), and 13 (case #1) (Figure 2D) (Table 1). It is noteworthy that the *APOE* allele status for cases 12 and 13 was 3/3, while that for case 11 was 2/3 (Table 1). The data indicated a significantly higher number of identified nApoECF-positive NFTs in the hippocampus of the *APOE* allele 3/3 cases in comparison to the 2/3 case (Figure 2D).

### Co-localization of the nApoECF antibody within NFTs in hippocampal sections of the Down’s syndrome brain

**Figure 3.**
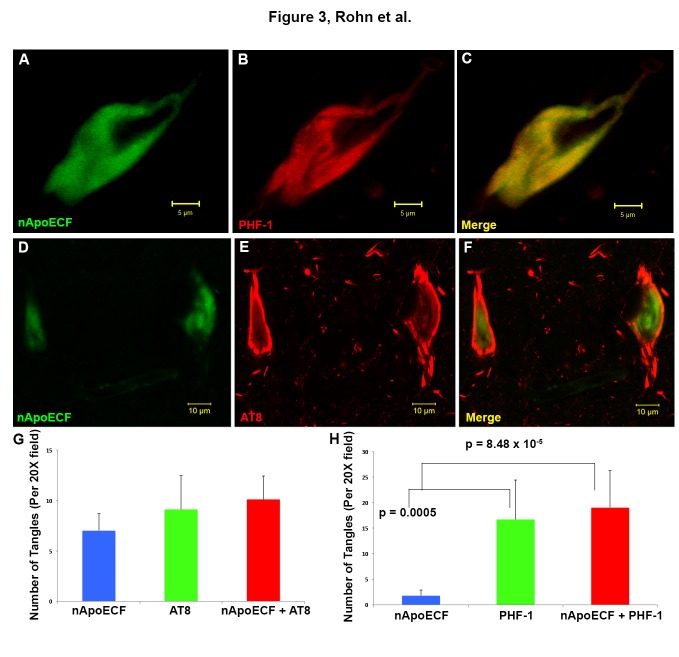
Co-localization of an amino-terminal fragment of apoE within NFTs in hippocampal tissue sections of the Down’s syndrome brain (**A-C**): Representative confocal immunofluorescence double-labeling utilizing the nApoECF antibody (green, Panel A) and PHF-1 (red, Panel B) revealed strong co-localization of the two antibodies within a NFT located in the hippocampus (Panel C). (**D-F**): Identical to Panels A-C with the exception of AT8 (red) being employed. For Panels A-F, images were captured from the CA1 region of the hippocampus in representative DS-AD cases. (**G and H**): Quantification of NFTs double-labeled by PHF-1, AT8, and nApoECF. For both panels, data show the number of NFTs labeled with nApoECF alone (blue bar), AT8 (**G**) or PHF-1 (**H**) alone (green bars) or those NFTs that were labeled with both antibodies (red bars). NFTs were identified in a 20X field within hippocampal tissue sections by immunofluorescence overlap microscopy (n=3 fields using three different DS cases) ±S.E.M. For Panel G there were no statistical differences between the various test groups. Data indicated that roughly 52.5% of AT8-positive NFTs also contained nApoECF, whereas 53.2% of all identified PHF-1-positive NFTs were labeled with nApoECF.

To determine the extent of co-localization of the nApoECF antibody, double-label immunofluorescence studies were performed using standard NFT tangle markers, PHF-1 and AT8 in fixed hippocampal brain sections. The antibody AT8 recognizes tau phosphorylated at both serine 202 and threonine 205, which are the first residues to be hyperphosphorylated, whereas the antibody PHF-1 recognizes phosphorylation at serine 396 and 404 and reacts with more mature hyperphosphorylated forms of tau found primarily within late-stage tangles [26-28]. Confocal immunofluorescence microscopy revealed strong co-localization between the nApoECF antibody and PHF-1 in the hippocampus (Figure 3A-C). Additionally, co-localization between nApoECF and the AT8 antibody was also evident, however, co-localization between the two appeared to be in distinct subcellular locations within the same tangle bearing neurons (Figure 3D-F). Quantification of NFTs double-labeled by PHF-1 or AT8 and nApoECF revealed that roughly 52.5% of AT8-positive tangle bearing neurons contained nApoECF (Pearson coefficient = -0.61), whereas 53.2% of all identified PHF-1-positive neurons were labeled with nApoECF (Pearson coefficient = 0.64) (Figure 3G-H).

### Co-localization of nApoECF with full-length amino terminal antibodies to apoE4 in NFTs of the Down syndrome brain

**Figure 4.**
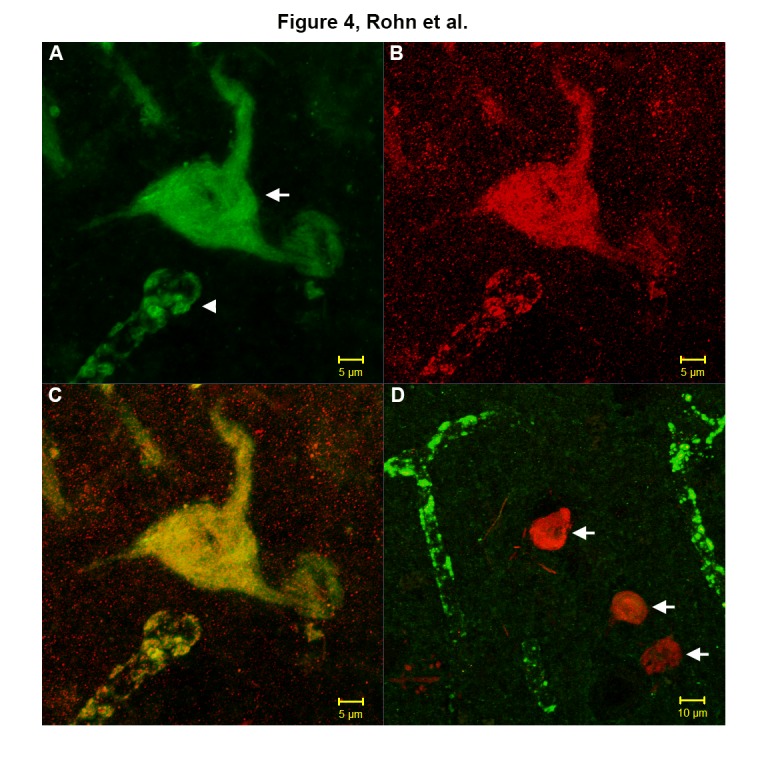
Co-localization of nApoECF with a full-length amino-terminal antibody to apoE4 in NFTs of the Down Syndrome brain (**A-C**): Representative confocal immunofluorescence double-labeling in CA1 region of the hippocampus in DS-AD cases utilizing an antibody that recognizes the amino-terminal region of full-length apoE4 (green, A) and nApoECF (red, B), with the overlap image shown in Panel C. Strong co-localization was indicated in both apparent NFTs and blood vessels (yellow, C). (**D**): In contrast, no co-localization with nApoECF was observed in a similar experiment using a full-length antibody to the C-terminal end of apoE4, which strongly labeled blood vessels (green, D), but not NFTs (arrows, D).

Further double-label immunofluorescence studies were performed utilizing an antibody specific to the amino-terminal region of full-length apoE4 and nApoECF (Figure 4A-C) in fixed hippocampal brain sections. Results showed strong co-localization within an apparent NFT (arrow, Panel C) as well as blood vessels (arrowhead, Panel C). By contrast, application of a full-length antibody specific for the C-terminal end of apoE4 strongly labeled blood vessels (Figure 4D, green) but resulted in a lack of immunoreactivity and co-localization with nApoECF within apparent NFTs (Figure 4D, arrows). The DS-AD cases used for these experiments are listed in Table 1 (11-13).

## DISCUSSION

Harboring the *APOE4* allele enhances the risk for AD and several reports have suggested that the proteolytic cleavage of apoE4 into N- and C-terminal fragments may provide a mechanism by which this protein contributes to AD pathogenesis (for recent review, see [21]). ApoE4 is highly susceptible to proteolysis compared to apoE3, and apoE4 fragments (14-20 kDa) have been identified in the AD brain [22, 29, 30]. However, whether apolipoprotein E4 contributes to an earlier onset of dementia or increased mortality in DS patients is still a matter of debate. Due to the location of the amyloid precursor protein on chromosome 21, many of the neuropathological features of early-onset AD including senile plaques and neurofibrillary tangles are also present in people with DS who are either demented or nondemented. Significant advances in medical treatment have increased longevity in people with DS resulting in an increased population that may be vulnerable to many of the same risk factors as those with sporadic AD. Few genetic risk factors associated with late onset AD carry a larger risk potential for AD than that of the *APOE4* allele. Inheritance of one copy of the allele increases risk four-fold while inheritance of two copies increases risk ten-fold [16]. The preponderance of evidence suggests that harboring the *APOE4* allele also increases dementia risk in DS, albeit to a lower extent than what is observed in AD [18]. Therefore, we sought to investigate whether apoE proteolysis is prevalent in postmortem DS human brain sections utilizing an in house antibody that detects the amino-terminal fragment of apoE. In AD this antibody, termed the amino-terminal apoE cleavage-fragment (nApoECF) antibody, predominantly labeled NFTs and co-localized with other tangle markers including AT8 and PHF-1 [22]. It is noteworthy that in AD, the nApoECF antibody is known to recognize fragmented E3 and E4 [22]. In the present study utilizing the nApoECF antibody, we examined fixed frontal cortex and hippocampal brain sections from seven adult DS-AD cases. An important caveat of the present study was the limited clinical information available regarding the *APOE* allele status of the patients used in this study. Therefore, we were unable to evaluate our findings in terms of *APOE4* status. Application of this antibody to frontal cortex tissues revealed labeling of mature NFTs as well as neuronal staining in DS-AD, while staining was restricted to pyramidal neurons in YDS cases. Not surprisingly, we did not observe labeling of NFTs in our YDS group as this is an age when NFTs are typically not present.

Interestingly, in addition to NFTs, the nApoECF antibody also labeled neurons in both frontal and hippocampal regions. In this regard, there was a significant difference in the number of labeled neurons in controls versus the DS-AD group and the YDS versus the DS-AD group. In general, labeled neurons morphologically appeared normal as compared to NFTs. In contrast, minimal immunoreactivity was observed in age-matched control cases. Results of experiments on hippocampal sections revealed little to no immunoreactivity in YDS and age matched control cases, and strong immunolabeling of NFTs as well as neuronal staining in DS-AD similar to what was seen in frontal cortex.

Another finding in the present study was the significant difference in the number of labeled nApoECF-NFTs in the hippocampus versus frontal cortex in the DS-AD group. There was significantly higher number of labeled NFTs in the hippocampus as compared to frontal cortex. This suggests the appearance of the fragmentation of ApoE coincides with the normal progression of NFT pathology from the hippocampus to the frontal cortex that is a well-characterized finding in AD [31]. Alternatively, the data could imply that in these three cases, there were substantially more NFTs present in the hippocampus as compared to the frontal cortex as this analysis was not performed. For these experiments, the *APOE* genotype of two of the three cases was 3/3, while that of the third was 2/3. The data indicated a significantly higher number of identified nApoECF-positive NFTs in the hippocampus of the 3/3 cases in comparison to the DS-AD 2/3 case (Figure 2D). These results would seem to support previous studies demonstrating that apoE2 is the most stable isoform [32] and may provide a rationale as to the protective role of harboring the *APOE2* allele in preventing dementia [33].

To further characterize the labeling of nApoECF within NFTs, double-label immunofluorescence confocal studies were performed using PHF-1 and AT8 antibodies. Results showed strong co-localization between the nApoECF antibody and PHF-1 as well as AT8 in hippocampal sections. With respect to AT8, our results suggested that even though the two antibodies co-localized within the same tangle bearing neurons, they appeared to be spatially separated. This was in contrast to PHF-1, where strong overlap with nApoECF was observed. In addition, statistical analysis revealed a negative correlation between AT8 and nApoECF (-0.61), while a positive correlation was found between PHF-1 (0.64). These data are suggestive that nApoECF accumulation occurs in more mature forms of NFTs and that the fragmentation of apoE is most likely a late event in the evolution of tangle pathology.

Double-label confocal immunofluorescence studies using an antibody specific to the amino-terminal region of full-length apoE4 and nApoECF showed strong co-localization in NFTs as well as blood vessels. By contrast, application of a full-length antibody specific to the C-terminal end of apoE4 resulted in a lack of immunoreactivity in NFTs but strongly labeled blood vessels. These results confirm our previous findings in AD, and support the specificity of the nApoECF antibody to the amino-terminal fragment of apoE4 [21]. In comparison of our previous study that examined late-onset AD cases we found a similar relationship between the presence of apoE fragmentation and its’ localization in NFTs of DS-AD cases. These data would suggest that the pathological events associated with apoE fragmentation in early onset-AD are similar when compared to late onset AD.

In summary, we have documented the presence of an amino-terminal apoE fragment in both the frontal cortex and hippocampus of the DS-AD brain. The presence of this fragment within NFTs of DS-AD brain tissue suggests these two events may be causally linked. Previous studies support that harboring the *APOE4* allele increases the risk for dementia in DS patients albeit to a lower extent to what has been found in AD. The present findings suggest that apoE fragmentation is present within the DS brain and could provide a rationale as to the enhanced dementia risk associated with the *APOE4* allele in DS. However, to best address this hypothesis, it would be necessary to compare autopsy cases with and without dementia, in addition to the presence or absence of AD neuropathology. Our data support a role for apoE proteolysis in DS-AD generating an amino-terminal fragment that accumulated with NFTs. Our study may provide impetus for further research on the potential impact of the *APOE4* risk factor on dementia in older DS populations.
